# Multicenter exploration of microbial communities in hospital toilets reveals: antibiotic exposure in nosocomial settings selects for *Enterococcus* over commensal taxa

**DOI:** 10.1186/s13756-025-01600-y

**Published:** 2025-07-01

**Authors:** Claudio Neidhöfer, Marcel Neuenhoff, Esther Sib, Anna Rehm, Lúcia Ribeiro Dias, Bernd Neumann, Jörg Steinmann, Manuel Döhla, Yousra Kherabi, Ana Budimir, Crystel Hajjar, Rouba Khatib, Katharina Axtmann, Katjana Schwab, Peter Brossart, Steffen Engelhart, Nico T. Mutters, Gabriele Bierbaum, Stefan Janssen, Marijo Parčina

**Affiliations:** 1 Institute of Experimental Hematology and Transfusion Medicine, University Hospital, Bonn, Germany; 2 Institute of Medical Microbiology, Immunology and Parasitology, University Hospital, Bonn, Germany; 3 Division of Clinical Bacteriology and Mycology, University Hospital Basel, Petersgraben 4, Basel, 4031 Switzerland; 4 Algorithmic Bioinformatics, Justus Liebig University, Giessen, Germany; 5 Institute for Hygiene and Public Health, University Hospital Bonn, Bonn, Germany; 6 Infectious Diseases Department, Unidade Local de Saúde São João, Porto, Portugal; 7 Faculty of Medicine, University of Porto, Porto, Portugal; 8 Institute of Clinical Microbiology, Infectious Diseases and Infection Control, Paracelsus Medical University, Nuremberg General Hospital, Nuremberg, Germany; 9 Department of Microbiology and Hospital Hygiene, Bundeswehr Central Hospital Koblenz, Koblenz, Germany; 10 Université Paris Cité, Inserm, IAME, Paris, France; 11 Department of Clinical and Molecular Microbiology, Clinical Hospital Centre Zagreb, Zagreb, Croatia; 12 Faculté de Pharmacie, Université Saint-Joseph, Beirut, Lebanon; 13 Department of Oncology, Hematology, and Rheumatology, University Hospital Bonn, Bonn, Germany

**Keywords:** Antimicrobial resistance, Hospital-acquired infections, Multidrug-resistant bacteria, Antibiotic exposure, Hospital sanitary facilities, Healthcare environment microbiome, Infection control

## Abstract

**Background:**

Excessive antibiotic utilization in hospital settings catalyzes the emergence and dissemination of multidrug resistant (MDR) bacteria, with sanitary facilities serving as critical vectors for their propagation. This study investigated the impact of patient antibiotic exposure on microbial diversity in hospital sanitary facilities, as well as the emergence of uniform communities and prospering taxa under antibiotic pressure.

**Methods:**

For this purpose a cross-sectional study was conducted between September 2022 and April 2023 from eight hospitals in seven cities across five countries, representing a diverse mix of tertiary care, military, oncological, psychiatric, and general teaching hospitals to analyze bacterial population differences in hospital toilets on wards with high versus minimal antibiotic administration using 16s rRNA amplicon sequencing.

**Results:**

PCoA analysis with Bray-Curtis and unweighted UniFrac metrics revealed microbial clustering influenced by antibiotic exposure and geography. Among all taxa analyzed, *Enterococcus* showed the strongest and most consistent association with high-exposure environments, making it one of the most striking findings in our dataset.

**Conclusion:**

Routine overuse of antimicrobial agents aimed at false patient safety promotes a high-risk environment in the sanitary facilities of respective wards. Hence, the issue of hospital acquired infections with MDR pathogens transcends mere pathogen spread, entailing significant changes to both environmental and microbial landscapes over time. The situation signals an emerging ecological problem within healthcare environments, and highlights the urgency for an integrated approach to antimicrobial stewardship. The low detection of key nosocomial Gram-negative genera likely reflects the focus on toilets rather than sinks or showers.

**Supplementary Information:**

The online version contains supplementary material available at 10.1186/s13756-025-01600-y.

## Introduction

Hospital-acquired infections and antimicrobial resistance are linked [1] and pose major challenges, particularly for immunocompromised patients who require extended hospital stays [2–4]. A cycle occurs when such patients receive antibiotics, which can decimate their microbial flora and create an environment where multi-drug resistant (MDR) bacteria thrive [1–3, 5]. Because of the large number of patients at high-risk for infections in hemato-oncology departments, antimicrobials are used to an inordinately high extent, and as a result are released in excessively large quantities through the patients into the same sanitary facilities [6–11]. Antibiotics excreted by patients into the wastewater system can significantly reduce the diversity of commensal and environmentally beneficial bacteria, thereby disrupting the microbial equilibrium. This selective pressure creates ecological niches that favor the survival and proliferation of multidrug-resistant nosocomial pathogens. Consequently, sanitary facilities in wards with high antibiotic usage can serve as reservoirs and amplification sites for these resistant organisms, facilitating their persistence and potential dissemination within the hospital environment [6–9]. The additional vigorous use of cleaning agents and disinfectants in these high-risk settings further exacerbates the problem by causing cross resistances when not performed adequately, e.g. exposure to sublethal concentrations of chlorhexidine or quaternary ammonium compounds has been shown to induce efflux pump expression and membrane adaptations that reduce susceptibility to carbapenems [12, 63–65].

Currently there is a lack of knowledge about the impact on the microbial community in terms of the present taxa, diversity and abundance of species. Our study’s primary aim was to examine the microbial taxa on hospital wards with different levels of antibiotic exposure. A critical aspect of our investigation was to explore the ecological ramifications of antimicrobial practices on hemato-oncological wards, where the use of prophylactic and therapeutic antimicrobials is highly prevalent. We hypothesized that such practices, although designed to protect patients from infections, might inadvertently engender homogenized microbial communities and high-risk microbial environments. The concern is that antimicrobials discharged into the patient’s immediate environment could cultivate multidrug-resistant niches that survive beyond the patients’ discharge, potentially serving as infectious reservoirs for future patients [6]. Furthermore, we aimed to investigate the resilience of non-pathogenic taxa in high antibiotic environments, considering the implications for horizontal gene transfer and resistance plasmid persistence [1, 9, 13].

Since toilets inevitably harbor a mixture of recently excreted microbes and more persistent biofilm-associated taxa, they offer a unique window into the intersection of patient-derived microbiota and environmental microbial persistence. By sampling toilet surfaces across wards with differing levels of antibiotic exposure, our aim was to capture this dynamic interplay and assess how ward-specific factors shape the microbial landscape at this clinically and ecologically relevant interface.

## Methods

### Preparation for sampling

DNAse- and RNAse-free sterile 4.5 ml Nunc CryoTubes (Thermo Scientific, Waltham, Massachusetts, USA) were filled with 3 ml eNAT medium (Copan, Brescia, Italy) under a sterile workbench and assigned a unique study number. We prepared 21 sample tubes for each participating center, ten of which were used to sample toilets in oncological wards, ten to sample wards with less antibiotic usage, and one negative control to screen for contaminants arising during transportation or handling together with 42 swabs (Copan, Brescia, Italy), two per tube. We also prepared an additional negative control to identify potential contaminants from the production process, shipping, or inherent to the products. We consistently followed the same sampling methodology across all centers. The toilet brush was screwed down the drain, slightly more firm than with conventional use. Next the toilet was flushed. After flushing, the brush was again used to brush the drain more vigorously without flushing, and then returned to its seat. Finally, two swabs were used to sample the point where the water surface and bowl meet all around, and the swab heads were broken into the designated test tube for that toilet. To ensure consistent sampling, as samples were collected by different individuals, participants received both written and video instructions, which can be found in the appendix, and all sampling materials were prepared in the medical microbiological research unit of the University Hospital Bonn (UKB) and shipped to the respective centers. Samples were collected between September 2022 and April 2023.

### Sample collection

The samples were collected from eight institutions located in seven cities across five countries, encompassing a diverse range of healthcare settings. These include the University Hospital Bonn in Germany, Bundeswehr Central Hospital in Koblenz, Germany, Klinikum Nürnberg in Germany, St. Joseph Hospital in Paris, France, both the Oncological Hospital and the Psychiatric Hospital in Porto, Portugal, the University Hospital Centre Zagreb in Croatia, and the Hotel-Dieu de France in Beirut, Lebanon. From each city, ten samples were obtained from toilets in wards with high antibiotic usage, and another ten from toilets in wards with minimal to no antibiotic usage. Low exposure wards were selected based on local knowledge of minimal antibiotic use, and centers were encouraged to confirm this through available prescribing information or consultation with their respective ABS representatives or clinical leads; psychiatry wards were suggested as potential examples, where applicable and meaningful within the specific institutional context. High exposure wards were selected in a similar manner, with hemato-oncological units proposed as a reference, but final selection was left to participating sites based on ward-level prescribing patterns and consultation with antimicrobial stewardship personnel. This resulted in a total of 70 samples for each setting.

### Sample processing and sequencing

As soon as sampling was completed in each center respectively, samples were sent back to the UKB within less than 3 days. At UKB, DNA was then extracted without interim freezing (on account of the eNAT medium) using the column-based PureLink Microbiome DNA Purification Kit (Thermo Fisher Scientific, Waltham, MA, USA) according to the manufacturer’s instructions within less than 7 days. At the end of the extraction process the DNA was eluted to 100µL volume and qualitatively and quantitatively evaluated using the NanoDrop OneC (Thermo Fisher Scientific, Waltham, MA, USA) and Quantus Fluorometer (Promega GmbH, Walldorf, Germany). Next, 16 S rRNA gene sequencing libraries were constructed using the Quick-16 S NGS Library Prep Kit (Zymo Research Europe GmbH, Freiburg, Germany) with its included optimized V1-V2 primer pairs. Samples were spread across three sequencing runs with each run containing samples from two or more centers. Each run included a positive control included in the kit, and a negative control. Rather than sequentially, samples were distributed randomly across the 96-well plate and to some degree across plates, to mitigate batch effects. For quantitative PCR, quality control, and normalization purposes the Bio-Rad CFX96 Real-Time PCR Detection System (Bio-Rad Laboratories, Inc., Hercules, California, USA) was used. After pooling, the DNA was quantified with the QuantiFluor dsDNA System on the Quantus Fluorometer (Promega GmbH, Walldorf, Germany) and diluted strictly according to the Illumina protocol for MiSeq sample preparation. For the final library a loading concentration of 12pM was chosen and a 10–15% Illumina v3 PhiX spike-in control added before running it on the Illumina MiSeq platform with 500cycle v2 and, due to stock availability, 600 cycle v3 Illumina MiSeq Reagent Kits. All reagents and equipment for sequencing of samples were obtained from Illumina, San Diego, CA, USA.

### Bioinformatics analysis

Paired-end reads were demultiplexed using Bcl2fastq-tool (v2.20.0.422). Most of the analysis steps were performed with QIIME2 (v.2023.2) [14]. Library adapters and primers were trimmed with Cutadapt (v4.4) [15], utilizing primers from the V1-V2 region FW: AGAGTTTGATYMTGGCTCAG; RV: GCTGCCTCCCGTAGGAGT as described by [53] for optimal trimming. Reads were denoised with Deblur (v.1.1.1) [16] to obtain ASVs of 150 nucleotides. Taxonomy for Deblur sequences was assigned via the q2-feature-classifier [54] of QIIME2 using the pre-trained Naive Bayes classifier which is based on full length ribosomal sequences of Greengenes2 [55]. As GreenGenes2’s taxonomy currently lacks labels for mitochondria and chloroplasts, we classified ASV sequences against the older GreenGenes (v13.8 [56]), database specifically ASVs assigned to ‘c__Chloroplast’ or ‘f__mitochondria’ as a pre-filtering. For detailed phylogenetic information, a SATé-enabled phylogenetic placement (SEPP) [18] tree was generated with the Greengenes SEPP reference (v13.8.99). Prior to statistical testing, a rarefaction step was conducted to standardize sample sizes, resulting in a rarefaction depth of 10,000 reads per sample. In addition, low abundant ASVs, i.e. less than ten reads in all samples together, were removed, resulting in a feature table with 20.142 features across 122 samples. PCoA ordination was performed with qiime2.plugins.diversity.methods.pcoa [20] and visualized using EMPeror (v1.0.3) [21]. All raw data is available at Qiita study ID: 14,921 (ENA follows). Statistical differences in microbial taxonomy were indeitfied with Ancom qiime2.plugins.composition.visualizers.ancom [52].

### Statistical analysis

Statistical analyses utilized Spearman’s rank correlation to assess the relationship between microbial taxa and ordinal variables such as geographic latitude and antibiotic exposure level (classified by ward type). Point-biserial correlation analysis was used to explore the relationship between specific microbial genera and antibiotic agents. The correlation coefficient (r) measures the strength and direction of association between variables. Two-tailed t-tests were employed to identify significant differences in taxa prevalence across different levels of antibiotic exposure settings, the t-value reflecting the magnitude of the difference relative to the variation in the sample data. p-values were Benjamini-Hochberg corrected for multiple testing.

### Data and ethics

For all high-risk wards information on the most frequently prescribed antibiotic agents were collected from the ward physicians or ABS representatives in order to check varying taxa prevalence also in this respect. The ethics committee of the University Hospital Bonn confirmed that no ethics approval was required for this study. In all centers permission to collect the samples was obtained from the relevant committee, institutional review board, or hospital board, if needed. All data relevant to the study are included in the article or uploaded as supplementary information.

## Results

Overall, the dataset included 122 samples with read counts exceeding 10,000, of which 12 had read counts over 100,000 (See Figure A5 in the appendix). We excluded 18 samples with less than 10,000 reads from further analysis due to rarefaction. Samples from high exposure wards included such collected on oncological (35), hemato-oncological (16), neutropenic (3) and intensive care wards (4). Samples from low exposure wards included such collected on psychiatry (50), plastic surgery (10), and cardiology wards (4). This allowed an additional subdivision of the wards into very high exposure settings (hemato-oncological, neutropenic and intensive care), high exposure settings (oncological), intermediate exposure settings (cardiology) and very low exposure settings (psychiatry and plastic surgery) [22–24].

### Center and exposure variations in bray-curtis and unweighted UniFrac PCoA analysis

Quantifying microbial community differences between samples using the Bray-Curtis or unweighted UniFrac metrics and subsequent dimensionality reduction via PcoA, did not reveal striking clustering (See Figure A1 in the Appendix). Examining the similarity of the microbiomes via analysis of average distance of Bray-curtis and unweighted-UniFrac metric showed a significant difference when comparing low and high exposure wards with Permanova (See Figure A2 and A3 in the Appendix). Getting into differences between cities, the plot shows highest inhomogeneous values for Bonn and Porto with weighted-UniFrac metric and lowest homogeneity for Beirut and Bonn with unweighted-UniFrac (See Figure A4 in the Appendix).

### Exposure influence in alpha-diversity analysis

**Fig. 1:**
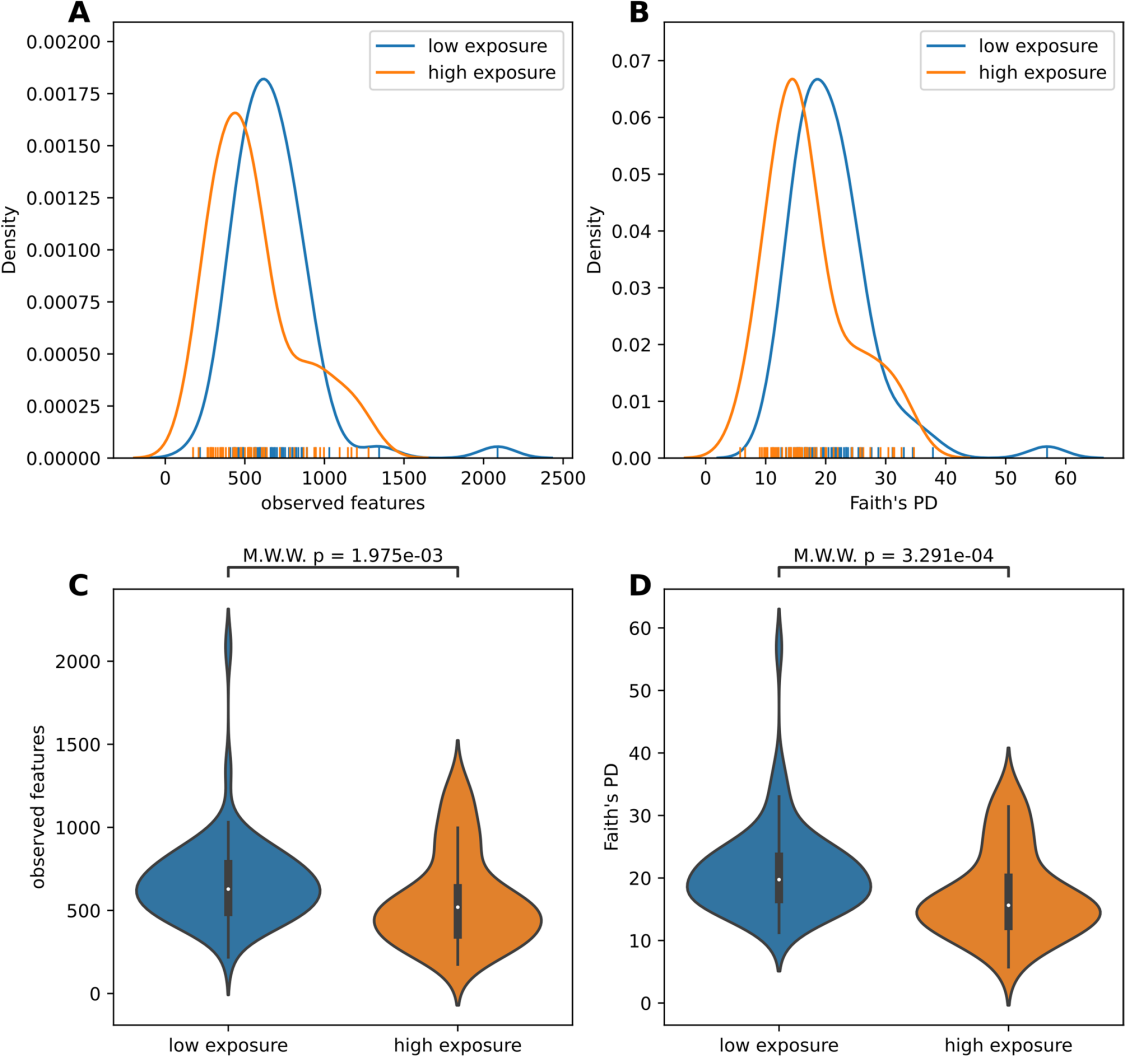
Density plots for observed features (**A**) and Faith’s Phylogenetic Diversity (**B**), with corresponding two-sided Mann-Whitney-Wilcoxon test results shown in (**C**) and (**D**), respectively

Looking into Alpha-Diversity revealed some significant differences between low and high exposure wards as well. The Mann-Whitney-Wilcoxon test for observed features indicates with p-value < 0.002 a significant difference for both ward types, the corresponding violin plot (Fig. 1C) shows a trend of generally higher microbial presence in low exposure wards. For phylogenetic diversity a p-value < 0.0004 with Mann-Whitney-Wilcoxon test (Fig. 1D) reveals significant difference between both ward types. Compared to the alpha-metric plot (Fig. 1A and B) low antibacterial exposure samples show a generally higher phylogenetic and evolutionary diverse community.

### General differences linked to the degree of antibiotic exposure

**Fig. 2:**
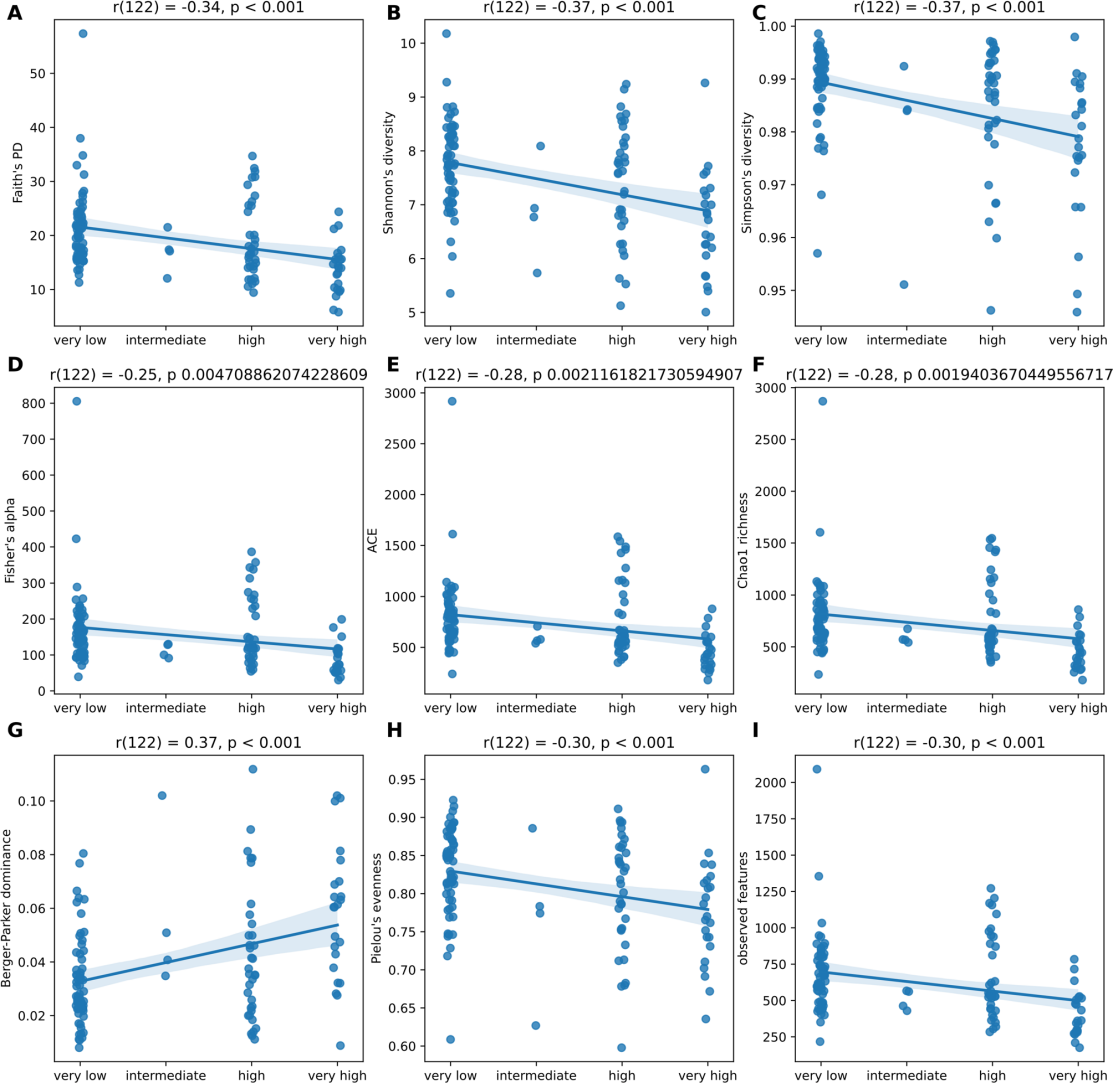
Scatter diagrams with regression line on the correlation between alpha diversity indices, evenness and richness and the degree of exposure to antibiotics on wards (1: very low exposure, 2: intermediate exposure, 3: high exposure, 4: very high exposure); **A**: Faith’s Phylogenetic Diversity, **B**: Shannon Diversity Index, **C**: Simpson Diversity Index, **D**: Fisher’s Alpha, **E**: ACE (Abundance-based Coverage Estimator), **F**: Chao1 Richness Estimator, **G**: Berger-Parker Dominance Index, **H**: Pielou’s Evenness, **I**: Observed Features (ASVs)

We found a moderate negative correlation between antibiotic exposure on wards (defined by the exposure setting) and the phylogenetic (r(122) = -0.34, *p* <.001), Shannon (r(122) = -0.37, *p* <.001), Simpson (r(122) = -0.37, *p* <.001), Fisher’s alpha (r(122) = -0.25, *p* <.005), ACE (r(122) = -0.28, *p* <.003), and Chao1 (r(122) = -0.28, *p* <.002) diversity indices. Confirming this, we found a moderate positive correlation between antibiotic exposure and the Berger-Parker Index (r(122) = 0.37, p = < 0.001). Also there was a moderate negative correlation between antibiotic exposure and Pielou’s evenness (r(122) = -0.30, *p* <.001) and observed features/ richness (r(122) = -0.30, *p* <.001) (see Fig. 2).

### Taxonomic differences linked to the degree of antibiotic exposure

Despite major taxonomic discrepancies across the different centers, there were notable differences associated with the degree of antibiotic exposure. Many taxa were negatively correlated with increasing antibiotic exposure and thus increasingly undetectable in high exposure settings. An exception, however, was the genus *Enterococcus*, whose relative proportion increased with higher antibiotic exposure. *Enterococcus* abundance across samples ranged from none to 4.57%, averaging 0.1% (Table 1).

**Table 1:**
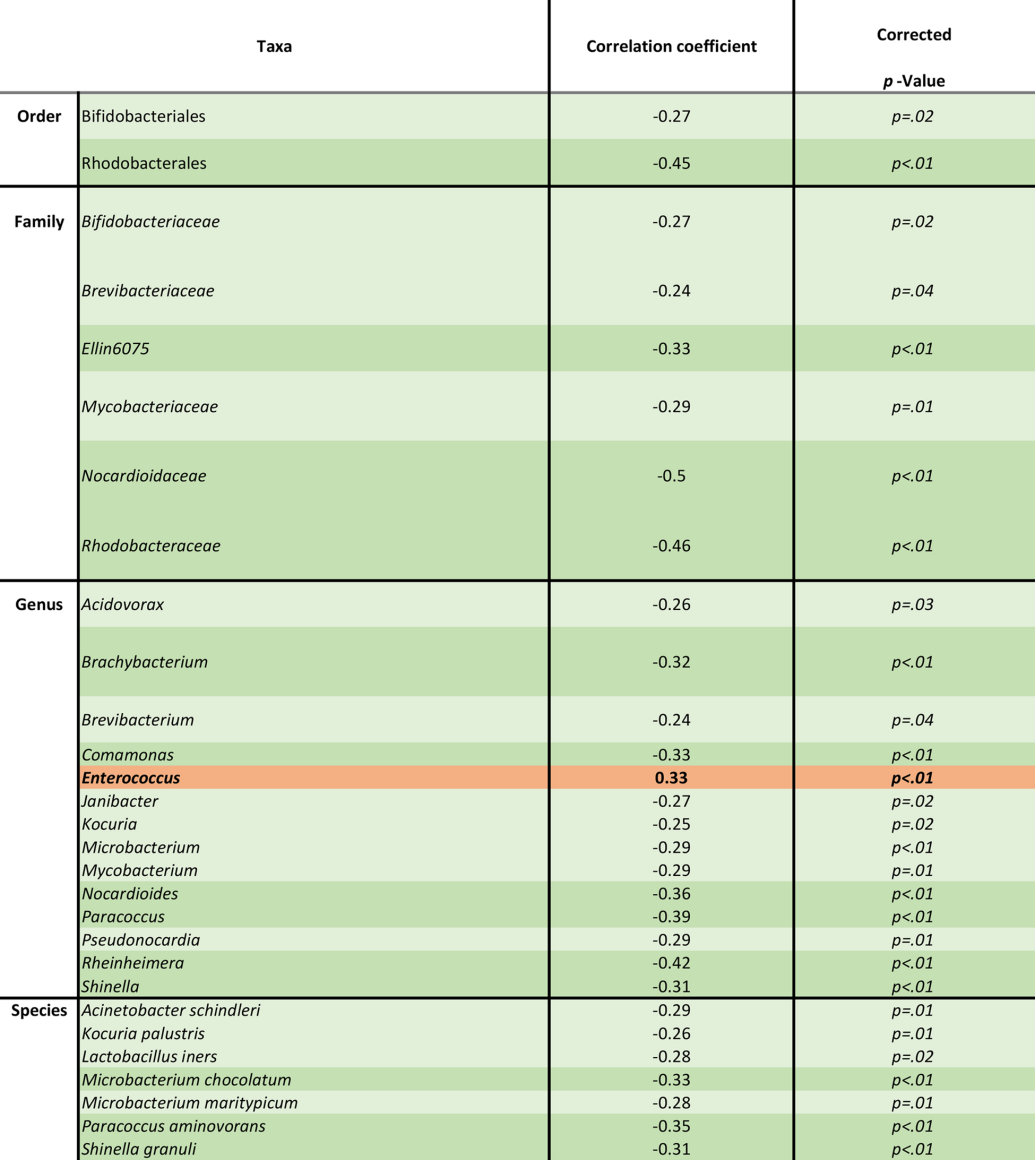
Results of spearman correlation between microbial taxa and antibiotic exposure levels, from very low to very high after Benjamini-Hochberg correction for multiple testing. Moderate negative correlation in green, weak negative correlation in light green, moderate positive correlation in red

## Discussion

The expected variability across different centers serves as a backdrop for the critical and robust association between the type of hospital ward and the corresponding patterns of microbial diversity and evenness. Irrespective of the inter-center differences, the amount of prescribed and subsequently excreted antimicrobials consistently influences the microbial populations in patient toilets, showcasing a clear pattern where the ward’s specific characteristics are mirrored in its microbial community structure [6, 25, 26].

The positive correlation of *Enterococcus* with antibiotic levels in hospital toilets supports anticipated outcomes, as important species of this genus such as *E. faecalis and E. faecium* have emerged as significant nosocomial pathogens, particularly due to their resistance to multiple antibiotics​ [27–32]. In fact, the widespread use of antibiotics in hospitals has been reported as key driver in the evolutionary shift, particularly of *E. faecium*, from a gut commensal to a proficient hospital pathogen [28, 29, 31]. This adaptability is enhanced by the acquisition of antibiotic resistance genes and traits that allow *Enterococcus* species to withstand environmental stressors, making them a persistent contaminant in hospital settings [31] and in particular, on wards with high-antibiotic exposure such as hemato-oncological ones [32]​.

*Enterococcus* species are known to thrive in a variety of environments, aided by their resistance to desiccation, heat, and disinfection, which ensures their survival in harsh hospital conditions [30]. The presence of antibiotic pressure in such environments and, in particular, the wide use of cephalosporins, further selects for resistant *Enterococcus* strains, allowing them to dominate over more susceptible microbial communities. This phenomenon is not only critical from an infection control perspective but also highlights the impact of antibiotics on the hospital’s environmental microbial landscape, as demonstrated by our study. The convergence of these factors emphasizes the complexity of managing *Enterococcus* as both an important nosocomial pathogen as well as a resilient environmental colonizer. It is important to note, however, that our sequencing approach does not resolve *Enterococcus* to species or strain level. The observed increase may thus reflect a mixture of taxa ranging from benign commensals such as *E. lactis* to clinically significant pathogens like VRE. As such, caution is warranted when interpreting the presence of *Enterococcus* as a direct proxy for nosocomial threat, though the consistent enrichment across high-exposure wards suggests that this niche is particularly favorable to *Enterococcus*, likely including those strains that pose a clinical risk.

Taxa that decrease with increasing exposure to antimicrobial substances can thus be explained by the opposite reasoning. *Lactobacillus* and *Bifidobacteriaceae*, widely recognized as beneficial commensals [33], are not particularly resilient to physical or antimicrobial stressors. Being more closely associated with the human microbiome, rather than with environmental biofilms such as those found in toilets [34], it is plausible to attribute their reduced detection primarily to the different patient populations. The demographics of oncological patients, often older with more comorbidities, could potentially introduce bias compared to psychiatry and plastic surgery patients. A subsequent study should aim to address this imbalance and consider including pediatric populations from both low and high exposure settings. This may also apply to *Enterococcus*, which might already have been selectively enriched within the patients in high-exposure settings themselves and antimicrobial pressure may have driven this selection even prior to environmental transfer. But while toilet biofilms inevitably contain a mixture of transiently shed material and more persistent microbial residents and the composition is undoubtedly influenced by the patient population, it is precisely this dynamic interplay between excreted microbiota and environmental persistence that our study aimed to characterize.

Given that our samples originate from toilets used by patients, the microbial signal likely represents a combination of human-associated and environmentally adapted taxa; for some genera, it remains unclear whether their presence primarily reflects patient shedding or environmental persistence, which is why known ecological functions, particularly in similarly stressed environments, warrant consideration in the interpretation of their enrichment or depletion. *Mycobacterium* species exhibit a dual role, with some species known for their pathogenicity to humans and others contributing to environmental bioremediation [35]. *Acidovorax*, *Brachybacterium*, *Brevibactrium*, *Janibacter*, *Kocuria*, *Paracoccus*, *Microbacterium*,* Nocardioides*,* Paracoccus*,* Pseudonocardia*,* and Shinella* are often found in aquatic environments, sludge and soil, respectively, where they play roles in nutrient cycling and pollutant degradation [36–40]. *Comamonas*, often associated with wastewater sludge and capable of surviving harsh conditions, including high levels of antimicrobials in industrial waste [41, 42], surprisingly was found negatively associated with increasing exposure in our study.

The use of antimicrobials for prevention and treatment, while crucial for patient safety, inadvertently applies selective pressure not only on the microorganisms within the host as the discharged residues from these antimicrobials also transform the surrounding environment into a breeding ground for multidrug-resistant organisms [6, 8]. Although patient sanitary facilities are frequently highlighted as key routes for spreading specific hospital pathogens [6–10], sometimes even more so than direct contact between patients or between patients and healthcare workers [43–46], the problem is more complex. It involves not just the dispersal of pathogens but also significant changes to the environmental and microbial community caused by everyday healthcare practices including room occupations and cleaning frequencies [47]. The relative absence of prominent nosocomial Gram-negative pathogens in our dataset likely reflects the sampling of toilet surfaces rather than other known hospital reservoirs such as sinks or showers, which have been repeatedly shown to harbor MDR Gram-negative organisms, including Pseudomonas aeruginosa and carbapenem-resistant Enterobacterales [26, 44]. Future studies should expand the environmental scope to include these high-risk areas to better capture the full range of clinically relevant resistomes.

Limitations of the study undoubtedly include the fact that actual antibiotic consumption across the different hospital wards was not assessed directly. Instead, the categorization into wards with varying levels of antibiotic use was based solely on literature from similar, albeit highly comparable, settings and on clinical experience. The collected data on antibiotic usage ultimately could not be meaningfully evaluated, as the heterogeneity in data collection methods across centers, ranging from informal estimates to partial pharmacy records, precluded reliable comparison or integration into the main analysis. Also, the study relied on 16 S rRNA amplicon sequencing, under the assumption that the detected taxa reflect, at least to some extent, the viable microbial communities present in the sampled habitat [61, 62].

Finally, it should also be considered that insufficient as well as too rigorous cleaning routines can promote cross-resistance and have been described as reducing diversity and raising MDR monocultures [48–51] and oncological high-risk wards are certainly subject to very rigorous cleaning interventions. In this context, probiotic cleaning may offer a more sustainable approach to managing microbial balance and addressing antimicrobial resistance [57–60], though its potential application in different wards and any associated considerations would need to be carefully assessed.

In our efforts to treat patients through antimicrobial use, we inadvertently foster environments where multidrug-resistant organisms thrive, extending beyond patient discharges to affect surrounding ecosystems such as ward sanitary facilities. These areas are increasingly recognized as significant conduits for the transmission of hospital pathogens, surpassing direct interactions between patients or between patients and healthcare workers. However, the challenge extends further than simple pathogen transmission. It involves a profound transformation of the environmental and microbial dynamics over time as a result of routine procedures.

## Conclusion

Our multicenter study demonstrates that antibiotic exposure is a key ecological force shaping the microbial communities of hospital sanitary facilities. Across diverse healthcare settings, we observed a consistent and robust enrichment of *Enterococcus* in high-exposure wards, suggesting that this genus thrives under selective antimicrobial pressure, likely reflecting both patient-derived and environmentally persistent populations. At the same time, several commensal and environmentally associated taxa showed marked depletion, indicating a loss of microbial diversity and resilience in these environments. This ecological simplification was further supported by significant shifts in alpha and beta diversity metrics, pointing to a narrowed microbial community structure under high antibiotic load. This evolution sets the groundwork for a wider ecological dilemma within healthcare environments, highlighting the urgent need for a holistic approach to antimicrobial stewardship.

## Electronic supplementary material

Below is the link to the electronic supplementary material.

Supplementary Material 1

## Data Availability

All data relevant to the study are included in the article or uploaded as supplementary information. All raw sequencing data is available at Qiita study ID: 14921. Sequence data that support the findings of this study have been deposited in the European Nucleotide Archive.
