# Supplementary material for: Multicenter exploration of microbial communities in hospital toilets reveals: antibiotic exposure in nosocomial settings selects for Enterococcus over commensal taxa
Source: Antimicrob Resist Infect Control. 2025 Jul 1;14:78. doi: 10.1186/s13756-025-01600-y (PMC12220145; doi:10.1186/s13756-025-01600-y)
Supplement: Supplementary file 1 — Supplementary Material 1 [file 13756_2025_1600_MOESM1_ESM.docx]

**Appendix**


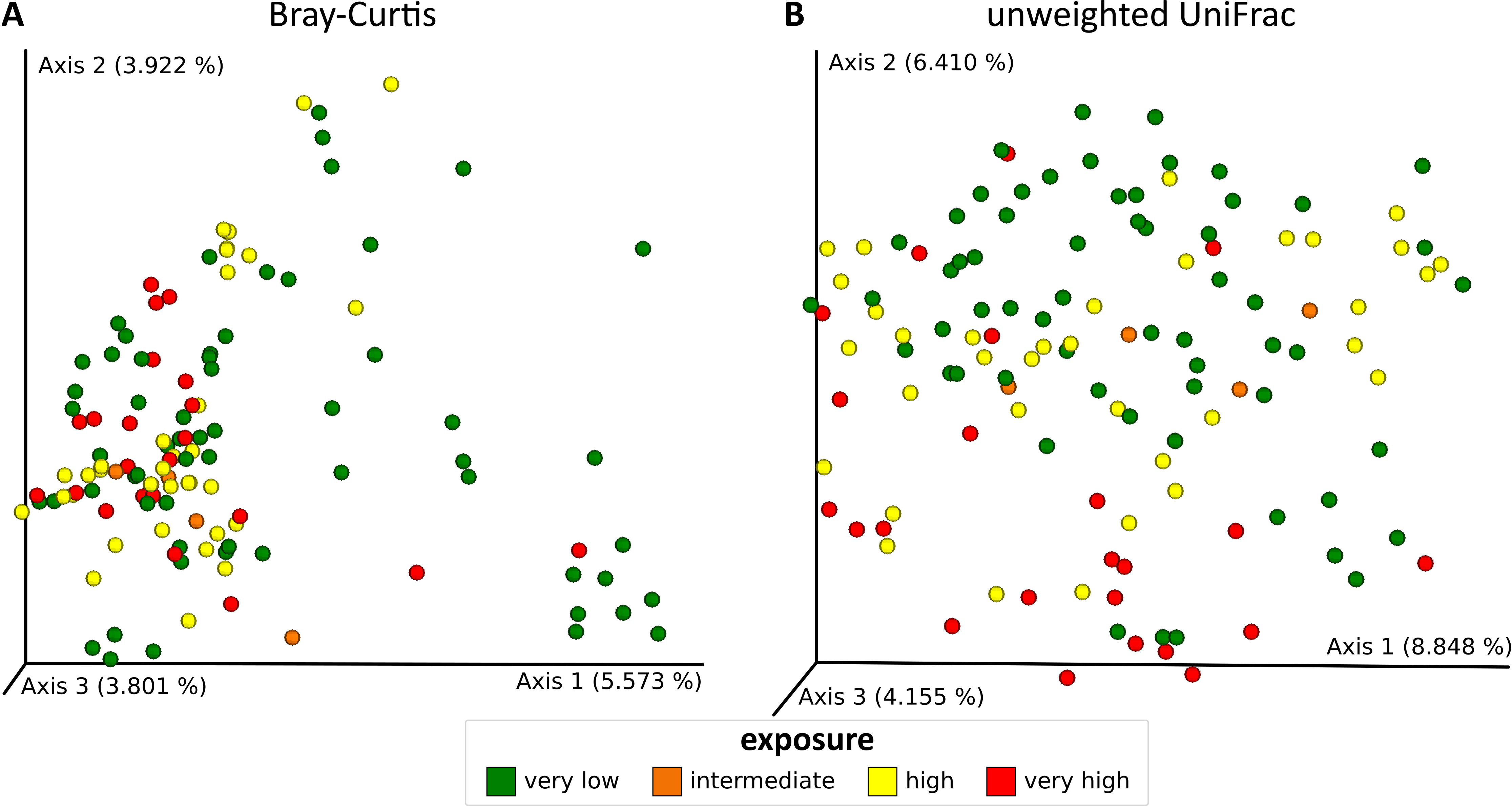


**Figure A1:** Principal Coordinate Analysis (PCoA) of Microbial Communities Based on Exposure Levels Using Bray-Curtis (A) and unweighted UniFrac Metrics (B).


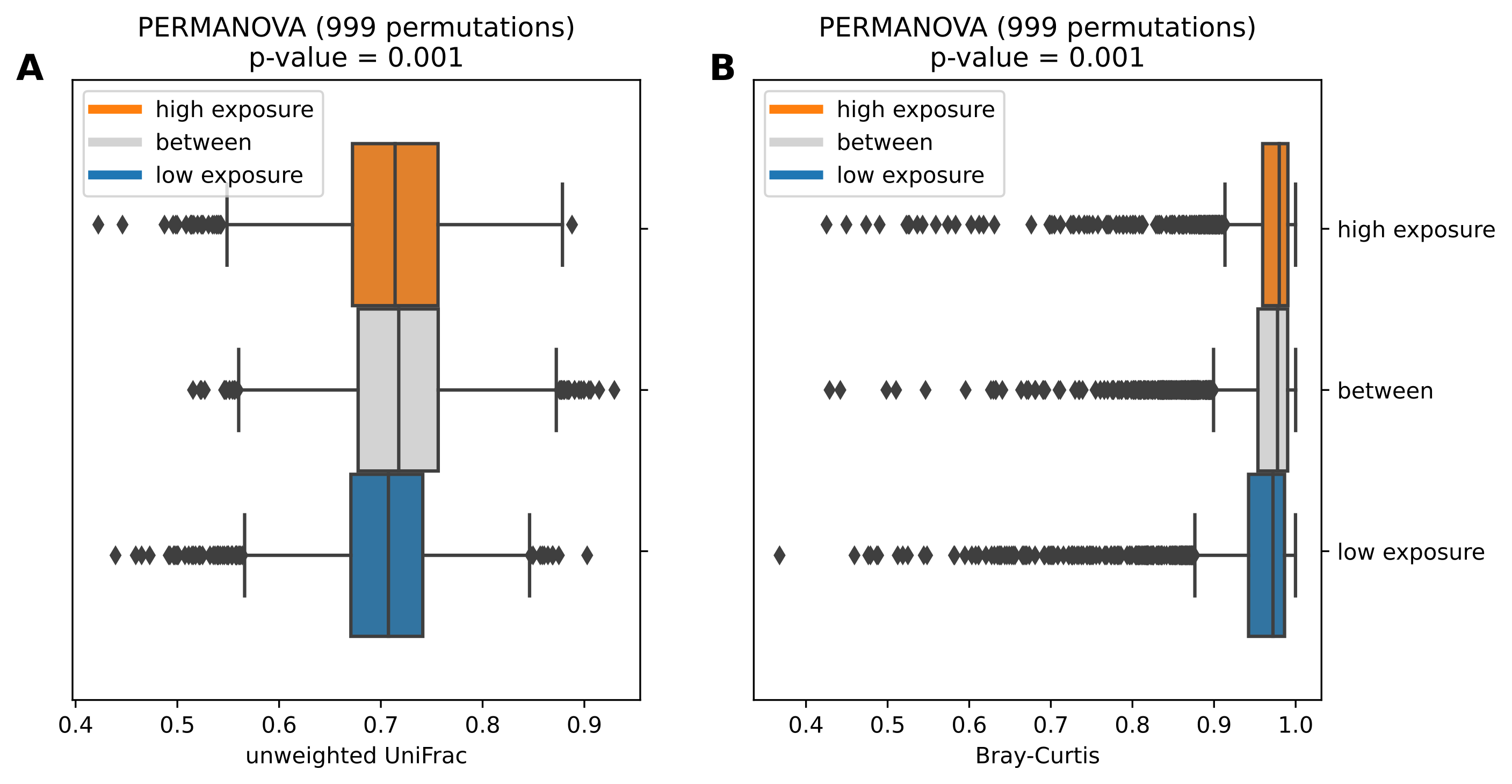


**Figure A2:** Pair-wise PERMANOVA test with 999 permutations for unweighted-UniFrac (A) and Bray-Curtis (B) metric showing significant difference with p-value: 0.001. Orange – high exposure, marine-blue – low exposure. Gray are distances between high-exposure and low-exposure samples.


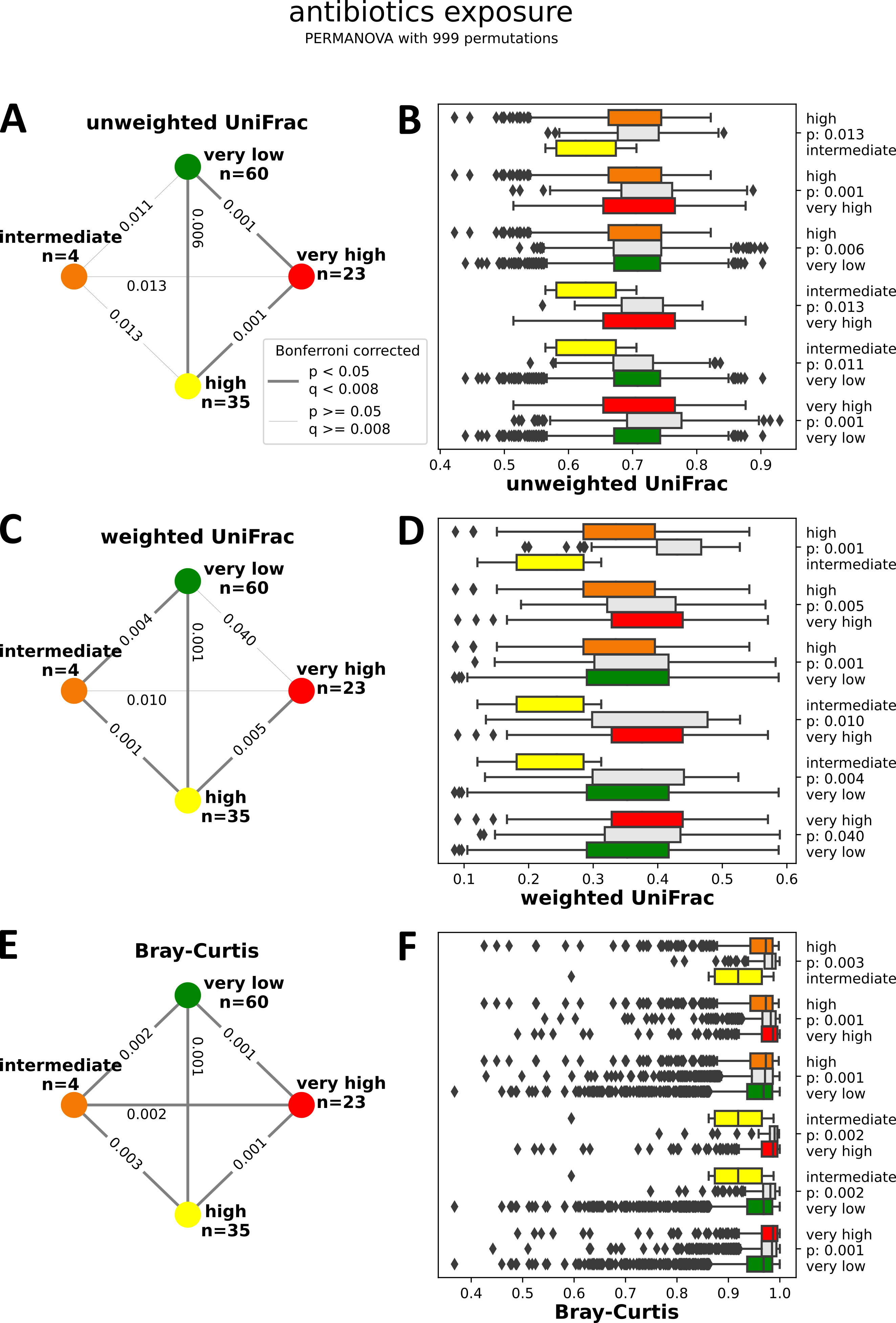


**Figure A3:** Beta Diversity Analysis of Microbial Communities Across Exposure Levels Using unweighted UniFrac (A, B), Weighted UniFrac (C, D), and Bray-Curtis (E, F) Metrics with PERMANOVA.


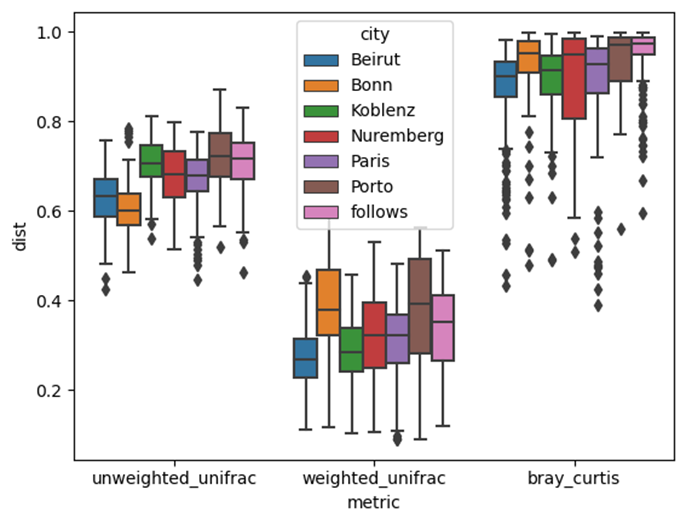


**Figure A4:** Homogenity plot for cities for Beta-Diversity metrics.


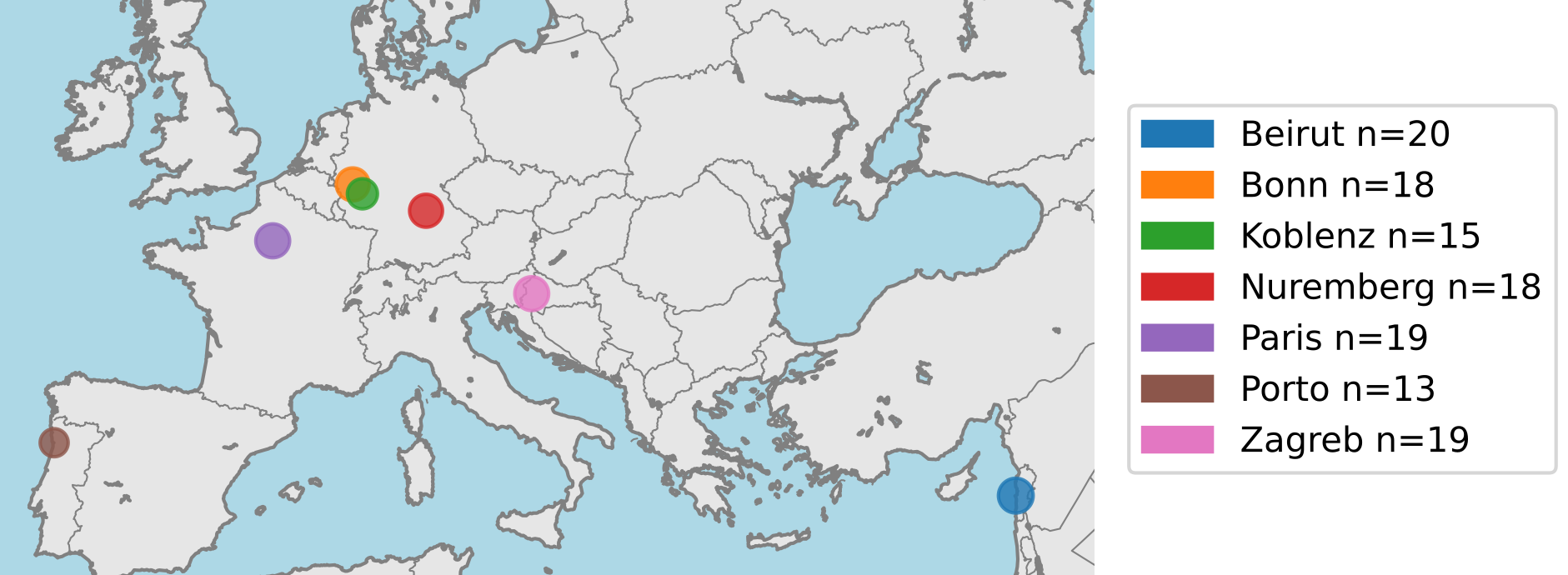


**Figure A5:** Centers and samples after rarefaction

**Sampling Instructions**

The toilet brush is placed inside the toilet. The toilet, containing the toilet brush, is then flushed. Once the flushing is completed, the toilet brush is used to brush the drain (much the same as with conventional use) but without it being flushed, and the brush returned to its seat. Finally, the point at which the water surface and the bowl meet is sampled in succession all around with two swabs, the heads of which are both broken into the one designated test tube for the toilet in question.

Video instructions found under: https://www.youtube.com/watch?v=KhJSHevPMOI
